# Conjunctival Intraepithelial Neoplasia Mimicking a Pigmentary Lesion in an HIV-Seropositive Indian Male

**DOI:** 10.7759/cureus.58953

**Published:** 2024-04-24

**Authors:** Jhimli Ta, Varsha Manade, Megha R Kotecha, Surbhi A Chodvadiya, Jessica Sangwan

**Affiliations:** 1 Department of Ophthalmology, Dr. D. Y. Patil Medical College, Hospital & Research Centre, Pune, IND

**Keywords:** ocular diseases of hiv, ocular surface squamous neoplasia (ossn), pigmented conjunctival lesion, conjunctival intraepithelial neoplasia, ocular oncology

## Abstract

We present the case of a 27-year-old male who presented to our ophthalmology outpatient clinic with a pigmented lesion on the conjunctiva of his right eye. There was no history of ocular trauma or familial ocular complaints, and a thorough evaluation revealed the patient's seropositive status for HIV for the past eight years. The presentation resembled a conjunctival pigmentary lesion, with typical features of ocular surface squamous neoplasia (OSSN) being absent and a demographic incongruent with typical OSSN cases as OSSN typically affects the elderly population. Given the patient's HIV status and the lesion's recent increase in size, a more aggressive treatment approach was warranted. Mass excisional biopsy surgery confirmed conjunctival intraepithelial neoplasia with one positive margin. Adjuvant treatment with mitomycin eye drops (0.04%) resulted in no lesion recurrence at the one-month follow-up. Conjunctival intraepithelial neoplasia can mimic pigmentary lesions in young HIV-positive patients with obvious signs of OSSN being absent. In such cases, the history of seropositivity should be sufficient to suspect it as OSSN and aggressive management measures should be adopted to get best possible outcomes.

## Introduction

Conjunctival intraepithelial neoplasia (CIN) also known as ocular surface squamous neoplasia (OSSN) is a dysplastic condition of the conjunctiva and is the most common tumor of the ocular surface. This condition is associated with various risk factors such as human immunodeficiency virus (HIV), human papillomavirus (HPV), ultraviolet light exposure, and petroleum products exposure. Conjunctival intra-epithelial neoplasia predominantly affects the elderly population; however, incidents of this ocular surface tumor occurring in younger individuals, particularly those who have tested seropositive for HIV have been reported in recent studies [1-3]. In this report, we describe the case of a 27-year-old seropositive (HIV) male of Indian origin presenting with a pigmented conjunctival lesion in his right eye. 

## Case presentation

A 27-year-old male of Indian origin presented with a complaint of a brown-colored lesion in the right eye for the past three years which was painless and non-progressive in nature. He noticed an increase in the size of the mass in the past eight months prior to his presentation. This was painless, gradual, and without any change in color. He also reported occasional mild discomfort and tearing in the same eye. He denied any significant decrease in vision, photophobia, or eye discharge. He reported good and stable vision in both eyes.

Past history revealed that he was diagnosed with HIV infection eight years ago and he has been on treatment for the same during the presentation with tab lamivudine+tenofovir combination once daily and tab atazanavir (300 mg)+ ritonavir (100 mg) combination once daily since eight years. The remainder of his history was unremarkable, with no other symptoms or systemic illness and allergies.

**Figure 1:**
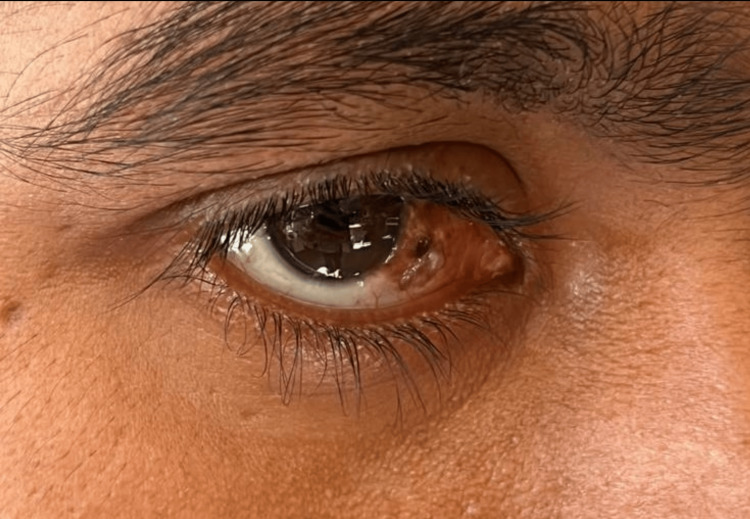
Pigmented, elevated, oval conjunctival lesion in the right eye

**Figure 2:**
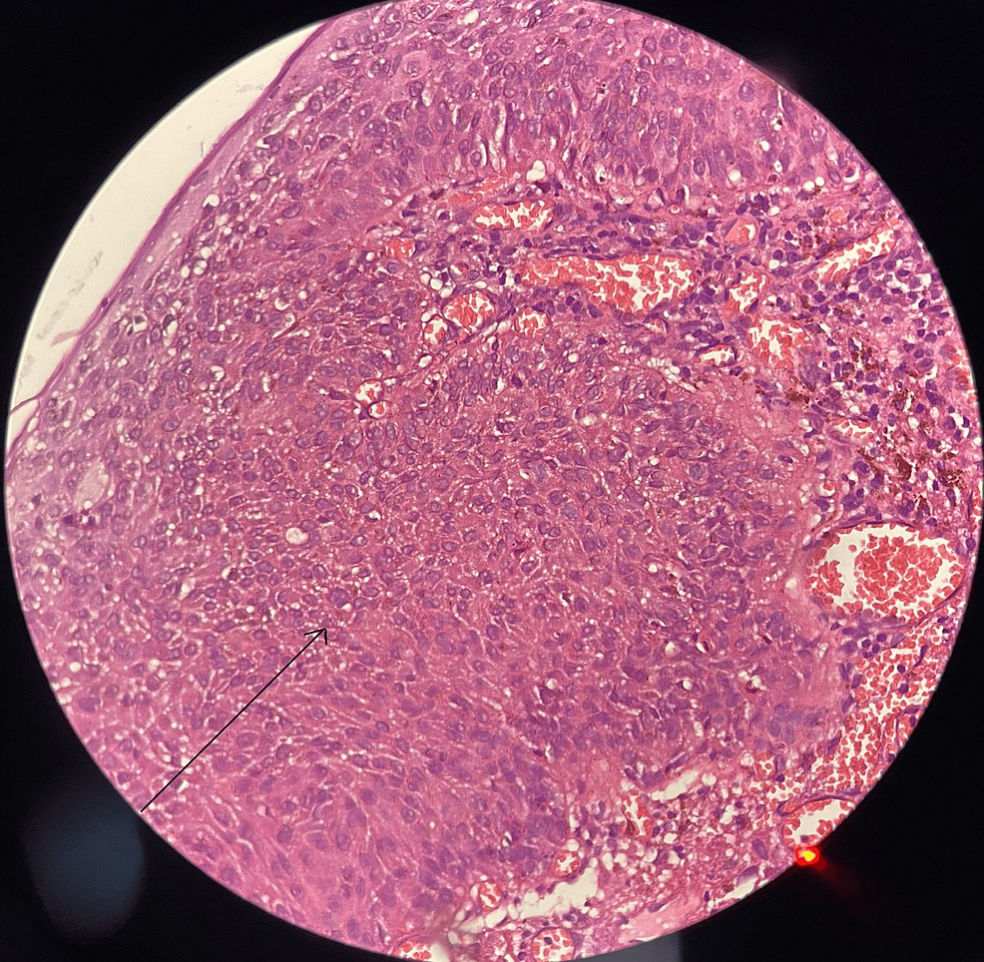
Histopathology of the lesion with hematoxylin and eosin (H&E) stain showing hyperplastic stratified squamous epithelium with nuclei enlargement, mild to moderate anisonucleosis, with loss of polarity and few prominent nucleoli (arrow)

**Figure 3:**
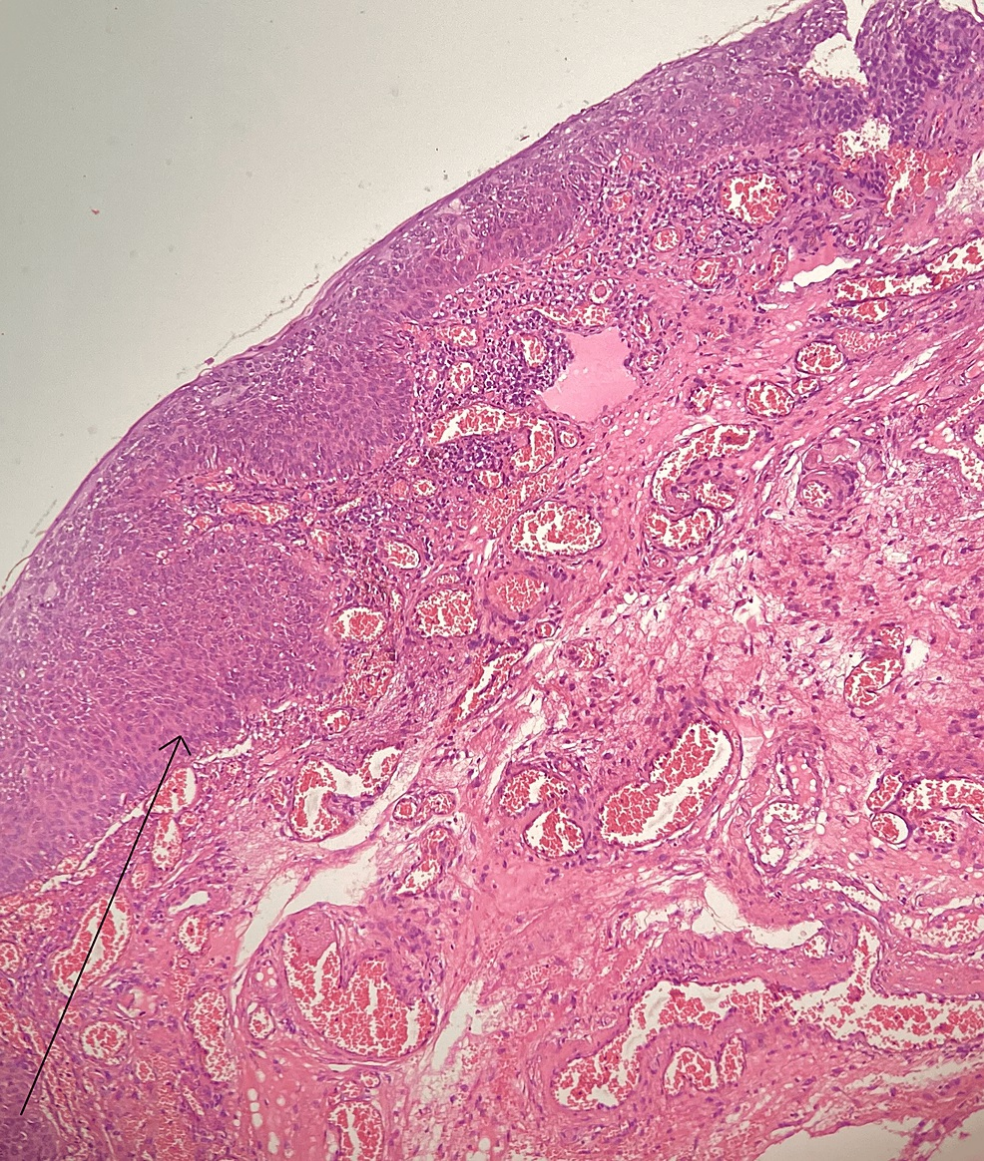
Histopathology of the lesion with hematoxylin and eosin (H&E) stain showing the atypical cells with enlarged nuclei, loss of polarity and prominent nucleoli (arrow)

Upon clinical examination, his best corrected visual acuity was 20/20 in both eyes. Posterior segment examination was unremarkable in both eyes. Anterior segment examination revealed a 14 mm x 10 mm, elevated, oval, pigmented lesion with irregular borders with vascularisation on the right nasal and bulbar conjunctiva in the right eye (Figure 1). HIV status was confirmed with serology. Since a strong association of OSSN has been in HIV-seropositive individuals, the patient was planned for a more aggressive treatment. A mass excisional biopsy was done with no touch technique to prevent tumor seeding. The histopathology report of the mass showed bits of tissue lined by hyperplastic stratified squamous epithelium with nuclei enlargement, mild to moderate anisonucleosis, with loss of polarity, and few showing prominent nucleoli. One end margin of the lesion showed evidence of CIN and the other end margin was free (Figures 2, 3). The histological findings of the lesion were consistent with CIN with one positive margin. The patient was treated with mitomycin C eyedrops 0.04%, one drop four times per day for 14 days, and he was advised to follow up after one month. The patient presented for the scheduled follow-up and no recurrence of the lesion was noted.

## Discussion

Conjunctival pigmentary lesions refer to a group of conditions of pigmentary lesions which can include various benign conditions such as nevi, primary acquired melanosis (PAM), and racial melanosis, or malignant conditions like melanoma. OSSN are invasive neoplastic lesions that affect the ocular surface, particularly the conjunctiva and cornea. OSSN includes a spectrum of lesions, ranging from mild dysplasia (CIN) to more severe forms such as carcinoma in situ and invasive squamous cell carcinoma (SCC). OSSN lesions may appear as areas of thickening, redness, or raised lesions on the conjunctiva and can sometimes mimic benign conditions initially, making early diagnosis challenging. Appearance can overlap, with some OSSN lesions initially resembling benign pigmented lesions, emphasizing the need for a thorough evaluation as OSSN has the potential to progress to invasive SCC, which can lead to significant vision loss and even life-threatening complications if not treated promptly. There are several well-established risk factors for CIN which include ultraviolet light exposure, HPV infection, immunosuppression (HIV/AIDS), a history of smoking, and petroleum product exposure. CIN predominantly affects the elderly population but there are incidents of this ocular surface tumor in younger individuals, particularly in HIV-positive individuals [1-3]. It is seen sometimes that OSSN is the first indicator of HIV infection with a significant number of people not knowing of their sero status until they presented with OSSN and were screened for HIV as evidenced by a study conducted by Kaliki et al. [4].

Clinically, CIN manifests as a fleshy, sessile, or minimally mobile growth, primarily located at the limbus. Less commonly, it may be found in the fornices and tarsal conjunctiva. These lesions can present with or without vascularization. CIN is typically categorized as either gelatinous or leukoplakic, the latter occurring when the lesion undergoes secondary hyperkeratosis. The recommended treatment for CIN encompasses active screening for early manifestations of OSSN, early and complete surgical excision, and close follow-up to address current high recurrence rates of 3-43% in HIV-positive OSSN patients [5].

Topical treatment with mitomycin C alone is effective in inducing regression of CIN in recurrent and surgically non-compliant cases [6]. Surgical removal of the lesion serves the dual purpose of eradicating the tumor and providing a definitive diagnosis [5]. However, surgical removal has its drawbacks, including the potential for residual cells on the conjunctival surface, an increased risk of recurrence, and a higher likelihood of conjunctival scarring and limbal stem cell deficiency.

Incisional biopsy is recommended for larger tumors that typically occupy more than four clock hours on the conjunctiva. This procedure involves removing part of the tumor, followed by appropriate therapy based on biopsy results. In contrast, excisional biopsy is reserved for smaller tumors (occupying less than four clock hours of the conjunctiva), symptomatic lesions, or those suspected to be malignant. This technique is employed to prevent inadvertent tumor seeding, a phenomenon where cancer cells spread to adjacent tissues along the biopsy needle's path [5]. Chemotherapy agents are often used in conjunction with surgical removal and may include Interferon alpha 2b (IFNa-2b), Mitomycin C (MMC), and 5-fluorouracil (5-FU) [7]. Mitomycin C, an alkylating agent, inhibits DNA synthesis and induces cell death via apoptosis and necrosis, with a preference for rapidly dividing cells [6]. It exhibits significant anti-tumor activity. A study by Blasi et al. indicated that adjuvant chemotherapy after surgery is strongly advisable over simple excision [7].

The risk and incidence of CIN in HIV-positive patients have been extensively studied in the literature. A study conducted in Kampala, Uganda, by Ateenyi-Agaba et al., revealed a significant increase in the incidence of SCC of the conjunctiva [8]. The incidence rate rose from approximately six cases per million per year in 1988 to 35 cases per million per year by 1992. The study evaluated all patients (n = 48) who presented with conjunctival SCC between February 1990 and February 1991, performing HIV tests on each individual. Remarkably, 75% of these patients were found to be HIV-seropositive, in contrast to only 19% of the controls. The study demonstrated that HIV infection conferred a 13-fold higher relative risk for the development of conjunctival tumors compared to the general population.

A study conducted by Waddell et al. investigated the association between HIV infection and conjunctival carcinoma, as well as the potential role of HPV-16 [9]. An increased frequency of CIN and invasive SCC was observed among patients with HIV-1 seropositivity. The study evaluated patients in Uganda and Malawi who presented to eye clinics with suspicious lesions for carcinoma. In Uganda, the study employed a case-control design, with two control subjects matched for age and sex for each case, sourced from the same health unit or residing in the same district as the cases. Pathological confirmation of the eye lesions was obtained through biopsies. HIV testing was performed on patients who underwent biopsy, as well as on the matched control subjects in Uganda. A sample of fixed biopsies was tested for the presence of HPV-16 using polymerase chain reaction (PCR) techniques. The study determined the HIV-1 serology, histopathological findings of conjunctival biopsies, and the prevalence of HPV infection. Conjunctival carcinoma was significantly associated with HIV infection, with an OR of 13.1 (95%CI 4.7-37.6). In Malawi, while there were no control subjects to calculate an OR, 78% of the cases were HIV-positive, compared to a 33% HIV-1 seroprevalence among antenatal women in the same hospital.

A study by Karp et al. conducted in Miami, Florida, United States) examined the medical records of patients diagnosed with CIN between 1991 and 1993 [10]. The primary focus of the study was on investigating the HIV status of patients under the age of 50 who were diagnosed with CIN. The researchers were able to contact and evaluate six out of the nine patients in this age group. Remarkably, three of these six patients (50%) were found to be HIV-positive. Based on these findings, the study concluded that HIV testing and counseling should be considered for patients younger than 50 years old who are diagnosed with CIN, given the potential association between HIV infection and the development of this ocular condition in this age group.

Kaimbo Wa Kaimbo et al. conducted a study in the Congo to further investigate the clinical characteristics of SCC and CIN in patients with AIDS [11]. The study reviewed the biopsy results of patients diagnosed with confirmed cases of SCC and CIN between 1994 and 1997. Among the patients reviewed, three had SCC and seven had CIN. The researchers found that while the clinical characteristics of SCC and CIN in their patient group were similar to those observed in immunocompetent individuals, these conditions occurred at a younger age and exhibited a more aggressive course in their patient population. Despite similarities in clinical presentation, Kaimbo Wa Kaimbo et al. reported that SCC and CIN manifested at an earlier age and displayed more aggressive behavior in patients with AIDS compared to immunocompetent individuals.

A study by Kiire and Dhillon identified HIV infection as a significant risk factor, leading to approximately a 10-fold increase in the risk of developing CIN, with most cases of CIN in Africa occurring in HIV-positive individuals [12]. Another study by Waddell and Newton provides further evidence of the etiology and associations of CIN, a precursor to invasive SCC of the eye [13]. Their findings reinforce the strong link between HIV infection and CIN, with HIV-positive individuals having a 13-fold higher risk of developing CIN compared to HIV-negative individuals. CIN typically impacts the elderly population. However, recent studies have documented occurrences of this ocular surface tumor in younger individuals, particularly those who have tested seropositive for HIV [1-3]. Cackett et al. reported a case involving a 38-year-old Zambian woman who presented with a conjunctival lesion that preceded her diagnosis of HIV infection by five years [2]. Initially, the lesion was presumed to be benign, and consequently, a conservative management approach was adopted. However, upon the subsequent diagnosis of HIV seropositivity, a biopsy of the lesion was performed, revealing it to be conjunctival intraepithelial neoplasia (CIN). This case highlighted the importance of considering the possibility of CIN in patients with conjunctival lesions, particularly in the presence of risk factors such as HIV infection [2].

Another study by Mondal et al. reported a case of CIN in a 38-year-old male who tested positive for HIV in Kolkata [3]. The patient presented with a growth at the limbus of his left eye, and the mass was excised under local anesthesia. Biopsy results confirmed the diagnosis of CIN, with serological tests confirming HIV positivity.

The treatment modalities of CIN and their effectiveness have been extensively studied. A study by Kiire and Dhillon suggested a low threshold for excision biopsy, which remains the treatment of choice for most patients, particularly in resource-poor settings, as topical chemotherapeutic agents and interferon require prolonged follow-up and may not be practical in developing countries [12]. Daniell et al. conducted a study examining the effectiveness of topical mitomycin C alone in treating corneal and CIN [6]. In their open prospective analysis, recurrent cases (n=17) and surgically non-compliant (n=3) cases of corneal and conjunctival intraepithelial neoplasia were treated with topical mitomycin C. Patients received mitomycin C eye drops, either 0.02% or 0.04%, four times daily for one week, followed by a week-long break, and then repeated for a second week. Weekly examinations were conducted until the lesions resolved, with clinical resolution observed in 18 out of 20 cases. The average time to resolution was 4.5 weeks, with an average of two treatment cycles administered. Their study showed that topical mitomycin c alone can be effective for regression and can be used for recurrent and surgically non-compliant patients.

Blasi et al. conducted a retrospective comparative study at the Ocular Oncology Service of the Catholic University of Rome between January 2006 and March 2016 [7]. The study included 79 patients with confirmed histological diagnosis of OSSN. Among them, 43 patients were treated with surgical excision alone (group A), 16 underwent surgical excision combined with topical mitomycin C (group B), and 20 underwent surgical excision along with adjuvant subconjunctival IFN-α-2b (group C). Recurrence rates varied across the three groups, with 31 recurrences (72%) observed in group A, 5 (31%) in group B, and 3 (15%) in group C. Their study underscored the challenges in managing OSSN with simple excision alone and highlighted the importance of adjuvant chemotherapy post-surgery to minimize recurrences. In Blasi et al.'s study, both interferon injections and mitomycin C drops demonstrated effectiveness in preventing recurrences and should be considered adjunctive therapies following surgical intervention.

To summarise, the preferred treatment of OSSN is surgical excision with adjuvant chemotherapy post surgery to achieve the least possible recurrence rate [7] but the treatment of choice for most patients, particularly in resource-poor settings, could be excision biopsy alone as topical chemotherapeutic agents and Interferon require prolonged follow-up which might not be conducive in developing countries [12]. For surgically non-compliant and recurrent cases of CIN, topical mitomycin C alone therapy can be employed as it has been shown to be efficient in causing regression in such cases [6].

## Conclusions

In cases of CIN mimicking conjunctival pigmented lesions, risk factors like HIV should be given prime importance to arrive at a provisional diagnosis and the management plan should be tailored accordingly as timely aggressive intervention is essential to preserve ocular health effectively. Early detection through meticulous anterior segment examination and HIV screening is essential, followed by prompt treatment.
